# Role of SiO_2_, TiO_2_, and Fe_3_O_4_ adsorbed on glycine for remediation of heavy metals and antibacterial activity in water

**DOI:** 10.1038/s41598-024-76285-1

**Published:** 2024-11-11

**Authors:** Noha M. Sabry, Rania Badry, Medhat A. Ibrahim, Hend A. Ezzat

**Affiliations:** 1 Water Pollution Research Department, Environment and Climate Change Research Institute, National Research Centre, 33 El-Bohouth St., Dokki, Giza, 12622 Egypt; 2 Center of Excellence for Research and Applied Studies on Climate Change and Sustainable Development, National Research Centre (NRC), 33 El Bohouth St. Dokki, Giza, 12622 Egypt; 3 Physics Department, Faculty of Women for Arts, Science and Education, Ain Shams University, Cairo, 11757 Egypt; 4 Spectroscopy Department, National Research Centre, 33 El-Bohouth St., 12622, Dokki, Giza, Egypt; 5 Molecular Modeling and Spectroscopy Laboratory, Centre of Excellence for Advanced Science, National Research Centre, 33 El-Bohouth St., 12622, Dokki, Giza, Egypt; 6 Nano Unit, Space Lab, Solar and Space Research Department, National Research Institute of Astronomy and Geophysics (NRIAG), Helwan, 11421 Egypt

**Keywords:** Glycine/Metal oxides composites, DFT, FTIR, Heavy metal removal, And Antibacterial agents, Environmental sciences, Materials science

## Abstract

Metals have a tendency to accumulate in the environment and can have carcinogenic effects. Accordingly, this study used density functional theory (DFT) calculations to investigate the adsorption of different metal ions on the glycine surface. Glycine has attracted a lot of research interest because of its remarkable metal-binding properties and cost effectiveness. Accordingly, to improve glycine’s adsorption capacity, it has been combined with SiO_2_, TiO_2_, and Fe_3_O_4_, creating a glycine-metal oxide nanocomposite through hydrogen bonding. After optimizing the structures at their energy minima at the B3LYP/6-31G(d, p) level of theory, the following analyses were carried out: total dipole moment (TDM), frontier molecular orbitals (FMOs), reactivity indexes, and molecular electrostatic potential (MESPs). The study of TDM, FMOs, reactivity indexes, density of states (DOS), and UV-Vis absorption analysis demonstrated the improved reactivity of glycine due to functionalization with SiO_2_. Additionally, the results showed that, compared to the glycine, the glycine/SiO_2_ surface experiences a greater degree of charge redistribution as a result of more hydrogen bonds being formed with adsorbate molecules. Thus, the study successfully extracted Cr, Fe, Co, Ni, Cu, As, Cd, and Pb from wastewater by demonstrating their selectivity for the glycine/SiO_2_ nanocomposite. The findings show that Ni had a stronger adsorption for glycine/SiO_2_ than the others as TDM increased (34.040 Debye), band gap energy decreased significantly (0.249 eV), and reactivity indices got improved. Additionally, the IR spectra were calculated and compared to the experimental data, which revealed remarkable frequency changes due to intermolecular interactions. HR-TEM scans validated the dispersion of SiO_2_ NP on the glycine surface with minimal aggregation. Furthermore, the antibacterial activity of glycine-amino acid-based surfactants was assessed, and the results show that glycine/SiO_2_ nanocomposites exhibited antibacterial efficacy against Gram-positive and Gram-negative microorganisms. These findings highlight the glycine/SiO_2_ nanocomposites for remediation of heavy metals and have antibacterial activity for treating pathogenic bacteria.

## Introduction

Fresh and pure water supplies are under threat everywhere in the world due to population growth and business expansion^1^. According to Al. et al. (1997), just 25% of the people living in the arena have access to freshwater^2^. Tariq and Mushtaq (2023) state that the main source of water contamination is man-made contaminants such as chemicals, fertilizers, industrial waste, pharmaceuticals, and other materials^3^. Furthermore, industrialized countries have the worst problems with chemical spills. Two million tons of industrial and sewage waste were taken out of the world’s water per day, according to a United Nations (UN) report^4^.

Minerals have a tendency to bioaccumulate in the body, and when consumed in excess of biologically recommended limits, they cause a wide range of biotoxic consequences^5^. Potentially hazardous metals include chromium (Cr), iron (Fe), cobalt (Co), nickel (Ni), copper (Cu), arsenic (As), cadmium (Cd), and lead (Pb) are the most common metal ions found in polluted water, whether from human activities or natural processes. These hazardous metals found in water can irritate the central nervous system and harm the liver^6^.

For example, chromium exists in nature in various oxidation states. Among its many global applications are pigments, metallurgy, catalysts, electroplating procedures, the tanning of textiles and leather, and wood preservation. Of the two stable forms of chromium found in water, Cr(III) and Cr(VI), Cr(III) is the least mobile, nontoxic, and involved in the metabolism of carbohydrates. On the other hand, Cr(VI) is very toxic and highly mobile in water. It causes mutagenic and teratogenic effects and forms Cr(V/IV) intermediates through intracellular reduction. The World Health Organisation (WHO) has set limits for Cr(VI) in wastewater that range from 5 to 500 µg L^−1^, and it recommends a maximum level of 50 µg L^−1 ^in drinking water^7^.

Iron in drinking water can cause issues, including reddish colour and odour. Fe in industrial water can cause corrosion of boilers and cooling lines, as well as damage to reverse osmosis membranes if not properly removed^8^. The World Health Organisation (WHO) recommends that iron concentration in drinking water be below 0.3 mg/L^9^.

The most hazardous heavy metal pollutants released into the aquatic environment are Co, Ni, Cu, and Pb. These pollutants are produced by the electroplating industry, phosphate fertilizers, batteries, stabilizers, pigments, and alloys^10^. They have been linked to a number of health risks for people, including the development of hypertension, insomnia, pain, anaemia, dizziness, irritability, muscle weakness, hallucinations, hepatic damage, renal damage, cancer, and central nervous system dysfunction^11^.

Arsenic contamination in wastewater, groundwater, and soil is a major issue in many countries, including China, Bangladesh, the United States, Chile, Taiwan, Mexico, Japan, Argentina, Poland, Hungary, India, and Canada^12^. Acute arsenic poisoning in humans can cause severe gastrointestinal problems, hepatic collapse, circulatory irregularities, or renal failure, as well as hyperpigmentation, cancer, and hyperkeratosis. Despite the existence of treatments for these acute symptoms, there are currently no therapies for chronic exposure; therefore, prevention is critical. According to recent reports, the most commonly used methods for As removal are precipitation, photocatalytic, oxidation, membrane filtration, coagulation, ion exchange, and adsorption^13^. Nonetheless, more work on developing safe and financially sustainable solutions for large-scale applications is required.

Cadmium, a bioaccumulative metal, ranks seventh on the Agency for Toxic Substances and Disease Registry’s toxic metals list. It is a non-essential and highly toxic metal that disrupts cellular enzymatic systems, causes oxidative stress, and leads to nutritional deficiencies in vegetables. Its toxicity directly affects the kidneys and liver. It can bind aspartate, histidine, cysteine, and glutamate, leading to iron deficiency. It is used in rechargeable batteries, special alloys, pigments, coatings, and as a plastic stabilizer, as well as in tobacco smoke. According to studies, Japan and China have the highest levels of cadmium exposure in the environment^14^.

Adsorption is one of the most practical, affordable, and widely used techniques for removing toxic metals from the aqueous environment^15^. A number of other common techniques have also been used to remove toxic metals from wastewater, including exchange of ions, chemical precipitation, complexation, extraction of liquid-liquid, reverse osmosis, the process of oxidation-reduction, evaporation, separation, adsorption, and Pulsed Laser Deposition^7,16^. All of these methods are expensive and inefficient, especially when heavy metal ions are present in wastewater at low concentrations. To overcome these drawbacks, alternative processes like adsorption have significant influence on removing toxic metals from wastewater^17^. Many natural materials possess adsorbent qualities and are available in large quantities. While numerous adsorbents occur naturally, cellulose, chitin, chitosan, wood, and coal have effectively been used to remove metal ions and dyes from wastewater^18–21^.

Glycine is the protein-forming amino acid, which is comparatively inexpensive, palatable, and approximately devoid of systemic side effects. Additionally, glycine is an α- amino acid (AA) with the chemical formula NH_2_CH_2_COOH. When it comes to protein formation, this most basic AA has the lowest molecular weight. It is also unique in that it lacks an asymmetrical carbon atom, which makes it non-optical and devoid of any L or D configuration. Glycine can adapt to hydrophobic and hydrophilic conditions inside the polypeptide chain thanks to its side chain hydrogen atom^22,23^. Since glycine is an important AA and has multiple functions for metabolites, a severe glycine deficit can have negative consequences on immunological response, cytoprotection, growth, and abnormal food metabolism^24^. Glycine is a primary component of extracellular proteins, including collagen and elastin. Furthermore, it has a critical function in the prevention of numerous illnesses and conditions like diabetes, cancer, and obesity^25^.

Glycine-functionalized magnetic nanoparticles (MNPs) have promising environmental applications, including the complexation and removal of harmful metal ions in water^26^. Magnetically separable and reusable MNPs, in instance, can remove more than 90% of Cu and Pb ions from water^27^. As a result, glycine-coated MNPs with precisely controlled structural and magnetic properties are critical for environmental, biological, and other applications^28^.

When acids protonate the amino group in the glycine chain, they create several cationic sites that increase the polarity and solubility of the material and improve its ability to absorb contaminants. Metals can particularly access the amine groups because they have electronic lone pairs on N atoms. Additionally, the electrostatic attraction between the adsorbent and anionic substances like halogens and anionic dyes is strengthened by this protonation. In addition, the presence of free OH and NH_2 _functional groups facilitates the adsorption of a wide range of pollutants, including heavy metals, phenols, pesticides, and antibiotics^23–25^.

As a result, much effort has been directed into the development of novel nano systems for the selective, rapid, and effective removal of harmful heavy metal ions from water^29^. Alswieleh et al. (2021) prepared mesoporous silica nanoparticles (MSNs) functionalized with three different functional groups (amine, iminodiacetic acid, and glycine). The adsorption efficiency of Pb and Cu is dependent on the carboxylic moiety present in the functional group; a twofold increase in removal efficiency is observed upon an increase in the carboxylic moiety^30^. Benettayeb et al. (2021) synthesized magnetic glycine-grafted chitosan for the sorption of Hg, Zn, and Ni with maximum sorption capacities of 0.35 m mol g^−1^, 0.47 m mol g^−1^, and 0.50 m mol g^−1^, respectively^31^. Hu et al. (2022) synthesized a new biodegradable chelator, methyl glycine diacetic acid (MGDA) to extract heavy metals from sewage sludge by chemical leaching. The maximum Zn, Cu, Ni and Cr leaching efficiencies of the synthesized MGDA were 94.1% ± 4.5%, 58.2% ± 3.1%, 78.2% ± 2.3%, and 54.6% ± 2.5%, respectively^32^.

The literature indicates that several experimental studies on the removal or recycling of heavy metals have been conducted, but theoretical studies on metal doping adsorption are insufficiently thorough. Additionally, the presence of bacteria in drinking water is a major cause for concern as it can lead to health outbreaks^33^. Therefore, eliminating bacterial contamination from drinking water is essential^34^. Accordingly, the first aim of the present work is studying the interactions of heavy metal atoms such as Cr, Fe, Co, Ni, Cu, As, Cd, and Pb with glycine/metal oxide nanocomposite surfaces based on density functional theory (DFT). Meanwhile, the second aim of this study is to assess the antibacterial activity of the synthesized glycine/SiO_2_ nanocomposites.

For these purposes, the influence of SiO_2_, TiO_2_, and Fe_3_O_4_ on the TDM, FMOs, reactivity descriptors, and MESP maps of glycine has been studied firstly. Moreover, the validity of the supposed model molecules was confirmed by FTIR spectroscopy, high resolution - transmission electron microscope (HR-TEM), and antibacterial activity.

## Experimental section

### Materials and instrumentation

Glycine, sodium silicate (Na_2_SiO_3_), sodium bicarbonate (NaHCO_3_), citric acid (C_6_H_8_O_7_), and hydrochloric acid (HCl) were purchased from Sigma-Aldrich. In this experiment, chemicals were utilized as-is, without any additional purification. The presence of SiO_2_ nanoparticles in the nanocomposites was characterized by Fourier-transform infrared (FTIR) spectrophotometer. The FTIR spectra were recorded using a FTIR-ATR spectrometer (Vertex 70, Bruker, Germany) starting from 4000 to 400 cm^−1^ at a resolution of 4 cm^−1^ for 35 scans. When combined with a type II alpha diamond crystal, the penetration depth of the diamond ATR attachment is 2 m. To test the background vs. air, the same parameters were applied at a resolution of 4 cm^−1^. The samples weren’t prepared before being used. The sample’s morphology was assessed using HR-TEM microscopy (JEOL, JEM-2010-F, Japan Electron Optics Laboratory co., Tokyo, Japan) at a 200 kV accelerating voltage. Using a vortex mixer, the particles were dissolved in ethanol before being examined on a gold grid (300 mesh, TED PELLA, INC).

### SiO2 and glycine/SiO2 nanocomposites preparation

In order to synthesize SiO_2_ nanoparticles, 1 M HCl was added, and the mixture was agitated at room temperature (RT) for 4 h after the precursor Na_2_SiO_3_ and distilled water (with fraction 1:2) were combined. This process continued until the pH changed from 12 to 7. The white powder was recovered by filtering, washing with distilled water to neutralize the acid, and drying for roughly 12 h at 60 °C. Add 1 equiv of silica gel 60 (230–400 mesh) to distilled water, then 2.1 equiv of NaHCO_3_, and stir at 50 °C for 1 h. Following the reaction, the synthesized glycine was added to the silica solutions at a concentration of 5% and agitated for approximately 1 hr. After gently cooling to room temperature, add citric acid dropwise to adjust the pH to 4.5^35^. The reaction accuracy is validated by FTIR and HR-TEM.

### Antibacterial activity of glycine/SiO2 nanocomposites

The in vitro antibacterial susceptibility of SiO_2_ NPs at 400 mg/ml and glycine/SiO_2_ nanocomposites (glycine-SiO_2_ NPs) has been evaluated against two bacterial strains: Gram-negative bacteria such as *Escherichia coli* (ATCC25922), and Gram-positive bacteria such as *Staphylococcus aureus *(ATCC 6538). The antibacterial activity of the synthesized compounds was assessed using the agar disc diffusion method, as described by Mukhtar et al. (2021)^36^. The bacteria were cultivated and swapped in Mueller-Hinton’s agar media, and discs of 6 mm diameter were made by punched Whatman filter paper. Single solutions of glycine-based SiO_2 _nanocomposites were prepared. After that, paper discs were submerged in the test nanocomposite solutions and placed on the plates containing bacteria. The plates were incubated overnight at 35–37 °C. Bacterial sensitivity to nanocomposites was assessed via measurement of the zone of inhibition (ZOI) in millimeters^37^.

### Calculation details

In our recent investigation, we used a glycine-metal oxide nanocomposite to remove heavy metals from water. Model molecules representing glycine, glycine/SiO_2_, glycine/TiO_2_, and glycine/Fe_3_O_4 _nanocomposite systems were subjected to calculations using the Gaussian 09^38^ program, which is used at the Molecular Modeling and Spectroscopy Laboratory, Centre of Excellence for Advanced Science, National Research Centre, Egypt. The Gauss View 05 program was used to visualize and draw molecular structures^39^. The Gauss Sum 3.0 program is used to plot the DOS spectrum^40^. Model molecules representing glycine and glycine/SiO_2_ were subjected to optimization at the B3LYP/6-31G (d, p) level of theory. The obtained values of the TDM and HOMO/LUMO bandgap energy are compared with those obtained by single point energy calculations. Table 1 presents the TDM and HOMO/LUMO bandgap energy for the optimized and single point energy calculations of glycine and glycine/SiO_2_. As presented in Table 1, the TDM of glycine is 1.246 and 1.310 Debye for glycine subjected to optimization and single point energy calculations, respectively. Meanwhile, for glycine interacted with SiO_2 _throughout the COOH functional group, TDM equals 6.368 and 7.087 Debye for the same sequence. Thus, since the results are approximately the same in addition to the fact that single point energy calculations are not time-consuming, all of the structures undergo single point energy calculations. Accordingly, the structures were evaluated for glycine/metal oxide adsorption with hydrated metals, including Cr, Fe, Co, Ni, Cu, As, Cd, and Pb. The proposed structure was subjected to single point energy calculations utilizing the DFT at the B3LYP/LanL2DZ level^41–43^. This B3LYP (Becke’s 3-parameter exchange functional with Lee-Yang-Parr correlation energy) hybrid functional includes the exchange-correlation functional, which is based on the standard form of the Vosko-Wilk-Nusair correlation potential^36^. The slater exchange was originally included in functional B, as were changes to the density gradient. Lee, Yang, and Parr developed a correlation functional LYP that incorporates both local and nonlocal variables^42,43^.

**Table 1:** Comparison between the optimization and single point energy calculations of the B3LYP/6-31G(d, p) calculated total dipole moment (TDM) as Debye and HOMO/LUMO bandgap energy ($\:\varDelta\:$E) as eV for glycine.

Structure	Optimization		Single Point Energy	
	TDM (Debye)	$\:\varDelta\:$E (eV)	TDM (Debye)	$\:\varDelta\:$E (eV)
Glycine	1.246	6.977	1.310	6.977
Glycine (COOH)-SiO_2_-H_2_O	6.368	6.563	7.087	1.298

To establish the adsorption interaction, an analysis was conducted that included TDM, FMOs, the energy difference between the highest occupied and lowest unoccupied molecular orbitals (HOMO/LUMO bandgap energy), global reactivity descriptors, MESP maps, and DOS. All of the calculated parameters were carried out utilizing the B3LYP/6-31G (d, p) model. Meanwhile, UV-Vis spectra were obtained through time-dependent density functional theory (TD-DFT) calculations. Electronic excitation energies were calculated using the Coulomb-attenuated functional (CAM-B3LYP). This functional provides better overall performance, as there is no correlation between the well-rounded, high-quality description of all excitation energy categories and excitation energy errors^44^.

The band gap energy plays an important role in determining the electrophilicity, chemical stability, and reactivity of chemicals. Electrophilicity index (ω), chemical hardness (η), and chemical potential (µ) were key parameters for analyzing charge transfer between metal and adsorbent. The equations below were used to analyze these parameters^45,46^.

1 $$\mathrm{Ionization}\;\mathrm{energy}\;\left(\mathrm{IE}\right)=-{\mathrm E}_{\mathrm{HOMO}}$$

2 $$\mathrm{Electron}\;\mathrm{affinity}\;(\mathrm{EA})\;=-{\mathrm E}_{\mathrm{LUMO}}$$

3 $$\mathrm{Energy}\;\mathrm{gap}\;(\Delta\mathrm E)={\mathrm E}_{\mathrm{HOMO}}-{\mathrm E}_{\mathrm{LUMO}}\;$$

4 $$\mathrm{Chemical}\;\mathrm{hardness}\;\left(\mathrm\eta\right)=\mathrm{Energy}\;\mathrm{gap}/2$$

5 $$\mathrm{Chemical}\;\mathrm{potential}\;\left(\mathrm\mu\right)=\left({\mathrm E}_{\mathrm{HOMO}}+{\mathrm E}_{\mathrm{LUMO}}\right)/2$$

6 $$\mathrm{Chemical}\;\mathrm{softness}\;(\mathrm S)=1/\mathrm\eta$$

7 $$\mathrm{Electrophilicity}\;\mathrm{index}\;\left(\mathrm\omega\right)=\mathrm\mu^2/2\mathrm\eta$$

8 $$\mathrm{Nucleophilicity}\;\mathrm{index}\;\left(N\right)=1/\mathrm\omega$$

9 $$\mathrm{Electronegativity}\;\left(\mathrm\chi\right)=-\mathrm\mu$$

10 $$\mathrm{Electronic}\;\mathrm{charges}\;\left(\Delta N_{max}\right)=-\mathrm\mu/\mathrm\eta$$

Finally, the theoretical and experimental infrared (IR) spectra were compared using the frequency computations (Opt-Freq) at their lowest energy. The theoretical IR were estimated using the DFT: B3LYP/6-31G(d, p) model.

## Result and discussion

### Building model molecules

**Figure 1:**
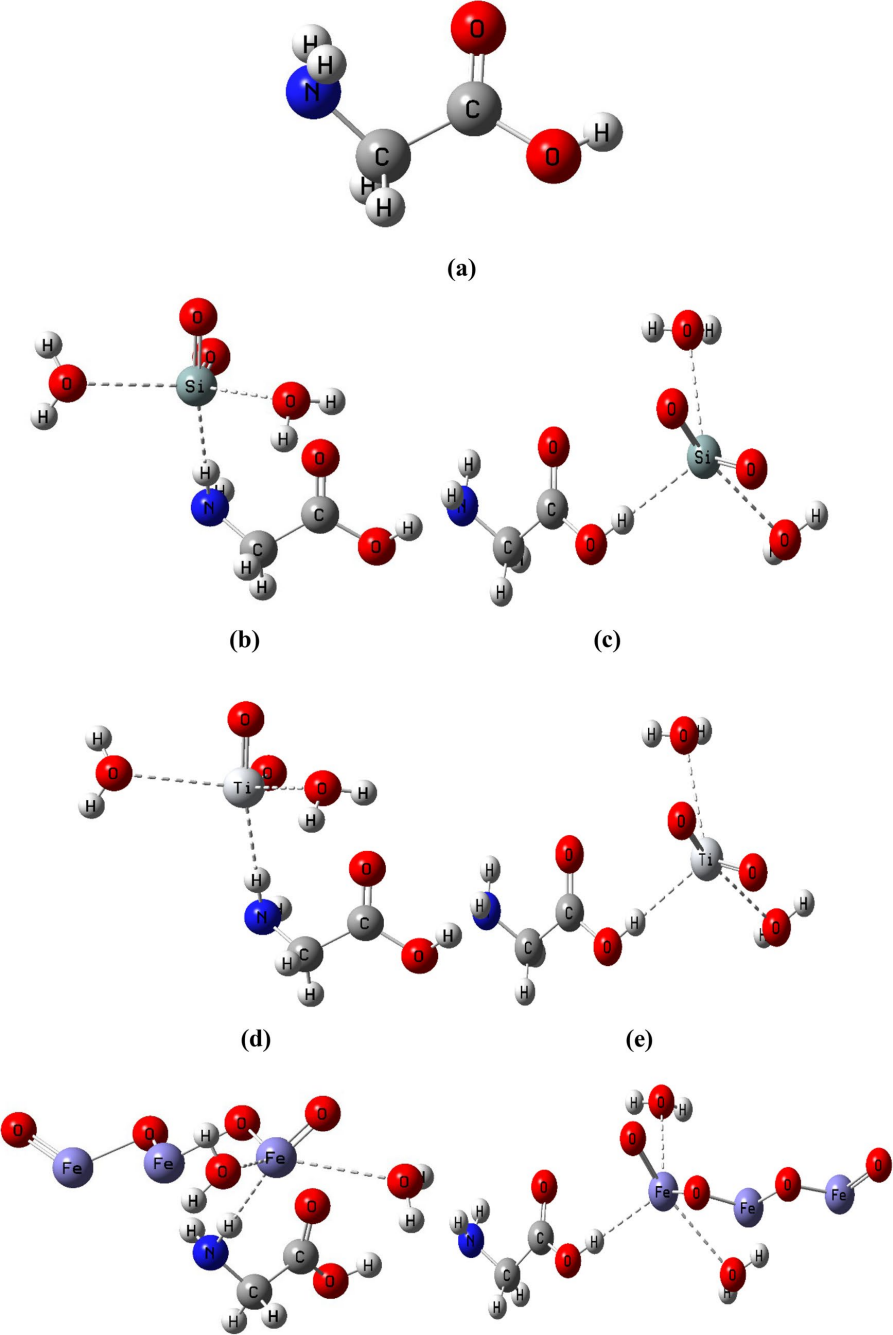
Model molecules of glycine two active sides (NH_2_ and OH) interacted with metal oxides including SiO_2_, TiO_2_, and Fe_3_O_4_ in presence of two water molecules.

Glycine, a well-known amino acid, has three active sites (NH_2_, OH, and = O), as shown in Fig. (1-a). One molecule of glycine is supposed to interact with one molecule of metal oxide. Metal oxides are likely to interact with glycine via the most active sides of contact, NH_2_ and OH. Furthermore, nanomaterials such as SiO_2_, TiO_2_, and Fe_3_O_4_ were chosen to study their effects on glycine properties and reactivity in the presence of two water molecules, as shown in Fig. 1- b, c, d, e, f, and g.

### Total dipole moment (TDM) for glycine/nano metal oxides model molecules

The change in the TDM, FMOs, and HOMO/LUMO bandgap energy were tracked to evaluate the influence of proposed nanometal oxides on glycine’s reactivity. Table 2 shows the TDM values for glycine, glycine/SiO_2_, glycine/TiO_2_, and glycine/Fe_3_O_4_ nanocomposite systems. TDM increased in response to interaction with the proposed metal oxides, indicating that functionalized glycine is more polarizable than pristine glycine. TDM increased from 1.310 Debye to 4.993 and 7.087 Debye for glycine interacted with SiO_2_ throughout the NH_2_ and COOH functional groups, respectively. Meanwhile, for glycine interacted with TiO_2_, TDM increased to 9.424 and 11.602 Debye for the interaction proceeds through the NH_2_ and COOH functional groups, respectively. Finally, for glycine interacted with Fe_3_O_4_, TDM increased to 14.135 and 14.106 Debye for the same sequence. The increased values of TDM of glycine due to the interaction with the studied metal oxides confirm the increased reactivity of glycine. Additionally, the variations in the TDM value of glycine caused by its interaction with SiO_2_, TiO_2_, and Fe_3_O_4_ confirm the establishment of hydrogen bonding between the hydrogen atom of glycine and the oxygen atom of the examined metal oxides. Moreover, the results confirmed that the most probable interaction between glycine and SiO_2_ and TiO_2_ proceeds through the COOH functional group. Meanwhile, for Fe_3_O_4_, the most probable interaction is that proceeds through the NH_2_ functional group.

**Table 2:** B3LYP/6-31G(d, p) calculated total dipole moment (TDM) as Debye and HOMO energy, LUMO energy, and HOMO/LUMO bandgap energy ($\:\varDelta\:$E) as eV for glycine/metal oxides including SiO_2_, TiO_2_, and Fe_3_O_4_ with two water molecules.

Structure	TDM (Debye)	E_HOMO_ (eV)	E_LUMO_ (eV)	$\:\varDelta\:$E (eV)
Glycine	1.310	-6.712	-0.583	6.977
Glycine (NH_2_)-SiO_2_-H_2_O	4.993	-5.715	-4.400	1.315
Glycine (COOH)-SiO_2_-H_2_O	7.087	-6.229	-4.931	1.298
Glycine (NH_2_)-TiO_2_-H_2_O	9.424	-4.656	-1.992	2.664
Glycine (COOH)-TiO_2_-H_2_O	11.602	-4.802	-2.668	2.134
Glycine (NH_2_)-Fe_3_O_4_-H_2_O	14.135	-5.321	-4.332	0.989
Glycine (COOH)-Fe_3_O_4_-H_2_O	14.106	-5.782	-4.646	1.137

### Frontier molecular orbitals (FMOs) for glycine/nano metal oxides model molecules

The two most significant orbital in a molecule are the LUMO and the HOMO orbitals. The biological activity of the molecule is reflected in the energy values of HOMO and LUMO as well as the energy gap between HOMO and LUMO^47^. HOMO/LUMO bandgap energy was used to investigate how the proposed metal oxides impact the reactivity of the glycine amino acid.

**Figure 2:**
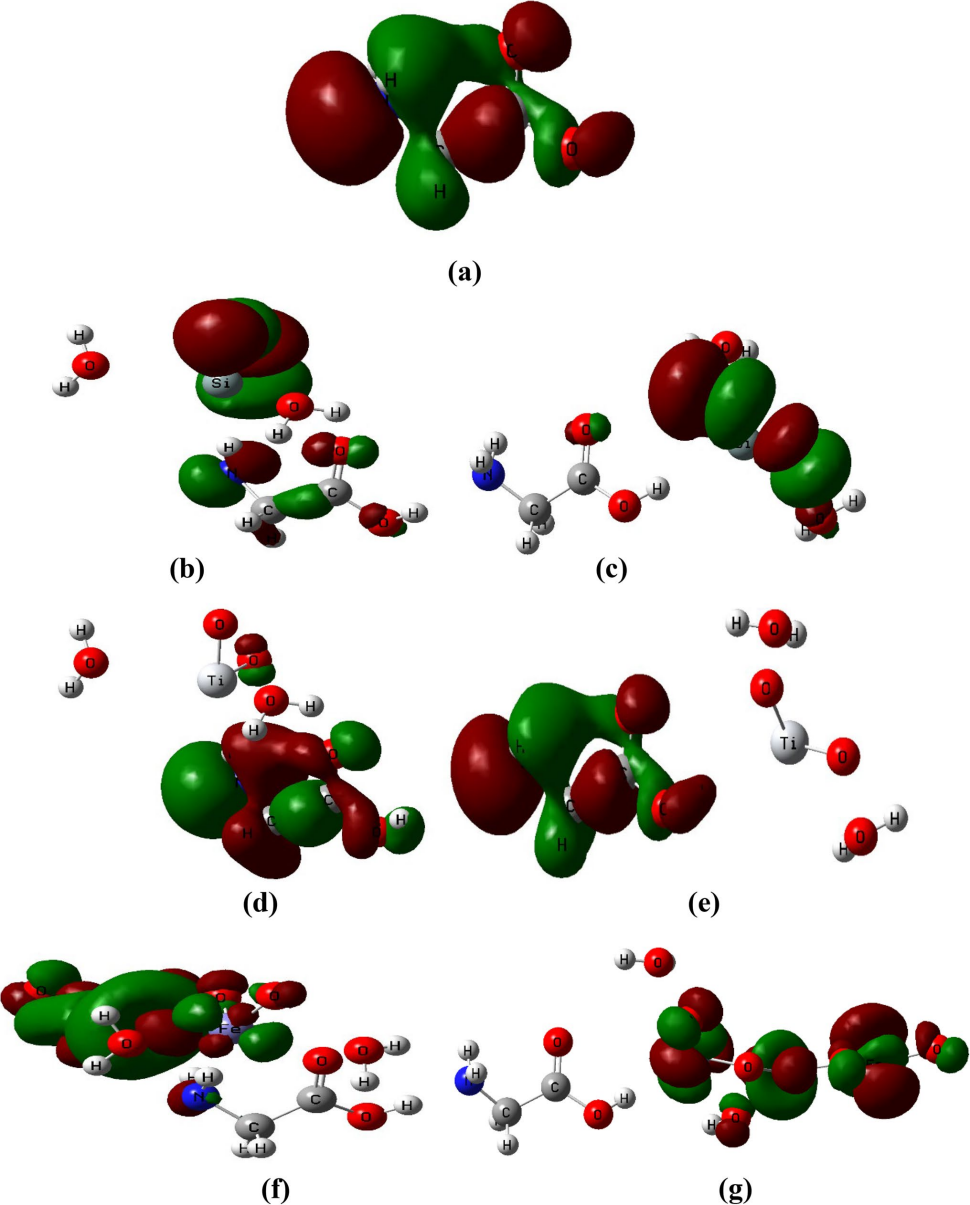
B3LYP/6-31G(d, p) calculated HOMO/LUMO orbital distribution of glycine two active sides (NH_2_ and OH) interacted with metal oxides including SiO_2_, TiO_2_, and Fe_3_O_4_ in presence of two water molecules.

Figure 2 shows the frontier molecular orbitals distribution of glycine, glycine/SiO_2_, glycine/TiO_2_, and glycine/Fe_3_O_4_ nanocomposite systems. The orbitals are dispersed uniformly around the glycine molecule, as illustrated in Fig. 2-a. However, the electronic charges are redistributed due to the interaction with the studied metal oxides (see Fig. 2-b, c, d, e, f, and g). In the case of glycine, the lone pair of N atoms and the π* charge of the carbonyl moiety had a highly centered charge distribution of the HOMO level. Additionally, the carboxyl group with the π* antibonding characteristic and the amino moieties of the glycine molecule showed a majority delocalization of the charge distribution of the LUMO level.

Different patterns in HOMO and LUMO energy levels appear during the upward transitions of intra-molecular charge transfer, as presented in Fig. 2. This makes it easier to predict different molecular properties by examining the ground state of the FMO. Charge transfer is caused by modifications in bonding properties, and charge transfer-related structural alterations can be effectively identified by the FMOs^48^.

Table 2 displays the computed HOMO, LUMO, and HOMO/LUMO bandgap energies for glycine, glycine/SiO_2_, glycine/TiO_2_, and glycine/Fe_3_O_4_ nanocomposite systems for both interaction mechanisms. It is obvious that the interaction of glycine with SiO_2_, TiO_2_, and Fe_3_O_4_ metal oxides has a significant impact on the glycine’s properties.

The results show that the HOMO energy of glycine increased from − 6.712 eV to -5.715, -4.656, and − 5.321 eV due to the interaction with SiO_2_, TiO_2_, and Fe_3_O_4_ throughout the NH_2_ functional group of glycine, respectively. Meanwhile, as the interaction proceeds through the COOH functional group, the HOMO energy increased to -6.229, -4.802, and − 5.782 eV. Which reflects the destabilization occurs in the HOMO energy.

On the other hand, the LUMO energy of glycine decreased from − 0.583 eV to -4.400, -1.992, and − 4.332 eV for the interaction proceeds through the NH_2_ group, while for the interaction proceeds through the COOH functional group, it decreased to -4.931, -2.668, and − 4.646 eV due to the interaction with SiO_2_, TiO_2_, and Fe_3_O_4_, respectively. This means that the LUMO energy of glycine stabilizes more due to the interaction with SiO_2_, TiO_2_, and Fe_3_O_4_. The observed increase in the HOMO energy and decrease in the LUMO energy reflect that intra-molecular charge transfer of electrons is stronger, thus the molecule’s reactivity increased.

Meanwhile, the HOMO/LUMO bandgap energy of glycine decreased from 6.977 eV to 1.315 and 1.298 eV, for glycine interacted with SiO_2_ through the NH_2_ and COOH functional groups of glycine, respectively. Meanwhile, for glycine interacted with TiO_2_, HOMO/LUMO bandgap energy decreased to 2.664 and 2.134 eV for the interaction proceeds through the NH_2_ and COOH functional groups, respectively. Finally, for glycine interacted with Fe_3_O_4_, HOMO/LUMO bandgap energy decreased to 0.989 and 1.137 eV for the same sequency.

The molecule is more polarizable due to the narrow frontier orbital gap, resulting in both low kinetic stability and strong chemical reactivity. Conversely, when glycine is compared to glycine/SiO_2_, glycine/TiO_2_, and glycine/Fe_3_O_4_ structures the comparison shows that the electronegativity increases and the energy gap decreases, which would lead to an increase in biological activity (see Fig. 2).

### Reactivity descriptors for glycine/nano metal oxides model molecules

Table 3 summarizes the computed global chemical reactivity descriptors of glycine, glycine/SiO_2_, glycine/TiO_2_, and glycine/Fe_3_O_4_ structures. These descriptors, including ionization energy, electron affinity, chemical potential, electronegativity, chemical hardness, chemical softness, electrophilicity index, nucleophilicity index, and electrophilicity, demonstrate the relationship between biological features and molecular stability.

The acquired IE values showed that the following order applies when removing an electron from HOMO to LUMO: As indicated in Table 2, Glycine > Glycine (COOH)-SiO_2_-H_2_O > Glycine (COOH)- Fe_3_O_4_-H_2_O > Glycine (NH_2_)-SiO_2_-H_2_O > Glycine (NH_2_)-Fe_3_O_4_-H_2_O **>** Glycine (COOH)-TiO_2_-H_2_O > Glycine (NH_2_)- TiO_2_-H_2_O. This means that IE decreased due to the interaction with SiO_2_, TiO_2_, and Fe_3_O_4_ through the two interaction mechanisms.

The probability of an atom acquiring an electron is known as electron affinity. An electron supplied to neutral atom releases energy, resulting in negative affinities. Because more energy is required to add an electron to a negative ion than is released during the electron attachment process, affinities are positive^46^. As presented in Table 2, EA increased due to the interaction with SiO_2_, TiO_2_, and Fe_3_O_4_ through the two interaction mechanisms. Additionally, EA follows this order: Glycine (COOH)-SiO_2_-H_2_O > Glycine (COOH)- Fe_3_O_4_-H_2_O > Glycine (NH_2_)-SiO_2_-H_2_O > Glycine (NH_2_)-Fe_3_O_4_-H_2_O **>** Glycine (COOH)-TiO_2_-H_2_O > Glycine (NH_2_)- TiO_2_-H_2_O > Glycine.

Another parameter to consider is the negative chemical potential (µ), also known as absolute electronegativity, which represents the transfer of electrons from a less electronegative system to a more electronegative system. Small values of global electronegativity indicate that electrons were delocalized on the molecule, allowing the molecule to easily provide electrons to coordinate suitable structure, resulting in greater bioactivity. In comparing to glycine/metal oxide structures, electronegativity has dropped, particularly for Glycine (COOH)-SiO_2_-H_2_O (-5.580 eV), but biological activity has increased.

Table 3 presents the alterations in the chemical hardness (η) and softness (S) of glycine subsequent to its nanocomposites with SiO_2_, TiO_2_, and Fe_3_O_4_. With these models, the chemical hardness dropped and the softness rose.

The results of small η values for the glycine and glycine/metal oxide nanocomposite systems represent the ability of charge transfer within the molecule^49^. As a result, the interaction between glycine and Fe_3_O_4_ and SiO_2_ has the lowest values of η. The linear relationship between η and HOMO/LUMO bandgap energy is seen in Tables 1 and 2. Regarding η values, the harder the molecule, the higher the η values, and the opposite is true for charge transfer^49^.

A crucial parameter for evaluating the tendency of a molecule to take electrons is its electrophilicity index (ω). One molecule behaves as an electrophile and the other as a nucleophile when two molecules come into contact. Following metal adsorption, the adsorbent’s electrophilicity increases indicating that the metal serves as an electron donor and the adsorbent as an electron acceptor, and vice versa for the nucleophilicity index (N). Additionally, the glycine/metal oxide composite’s ω rose, suggesting that the adsorbent’s adsorption capability was improved.

Another important parameter in studying the reactivity of biomolecules is the electronegativity (χ). The results presented in Table 3 confirmed that the χ of glycine increased due to the interaction with SiO_2_, TiO_2_, and Fe_3_O_4_. Finally, the electronic charges in glycine increased strongly as a result of the interaction with SiO_2_, TiO_2_, and Fe_3_O_4_. According to Table 3, the increasing order of χ is as follows: Glycine (COOH)-SiO_2_-H_2_O > Glycine (COOH)- Fe_3_O_4_-H_2_O > Glycine (NH_2_)-SiO_2_-H_2_O > Glycine (NH_2_)-Fe_3_O_4_-H_2_O **>** Glycine (COOH)-TiO_2_-H_2_O > Glycine (NH_2_)- TiO_2_-H_2_O > Glycine. All the results confirmed the increased reactivity of glycine due to the interaction with the studied metal oxides, which dedicate it to many applications.

**Table 3:** B3LYP/6-31G(d, p) calculated: ionization energy IE, Electron affinity (EA), Chemical potential (µ), Chemical hardness (η), Chemical softness (S), Electrophilicity index (ω), nucleophilicity index (N), Electronegativity (χ), and electronic charges (ΔN_max_) of glycine interacted with SiO_2_, TiO_2_, and Fe_3_O_4_.

Structure	IE(eV)	EA(eV)	µ(eV)	η(eV)	S(eV)^−1^	ω(eV)	(*N*)(eV)^−1^	χ(eV)	ΔN_max_
Glycine	6.794	-0.183	-3.306	3.488	0.287	1.566	0.639	3.306	0.948
Glycine (NH_2_)-SiO_2_-H_2_O	5.715	4.400	-5.057	0.658	1.521	19.448	0.051	5.057	7.691
Glycine (COOH)-SiO_2_-H_2_O	6.229	4.931	-5.580	0.649	1.541	23.984	0.042	5.580	8.596
Glycine (NH_2_)-TiO_2_-H_2_O	4.656	1.992	-3.324	1.332	0.751	4.148	0.241	3.324	2.495
Glycine (COOH)- TiO_2_-H_2_O	4.802	2.668	-3.735	1.067	0.937	6.538	0.153	3.735	3.501
Glycine (NH_2_)-Fe_3_O_4_-H_2_O	5.321	4.332	-4.826	0.495	2.022	23.547	0.042	4.826	9.758
Glycine (COOH)-Fe_3_O_4_-H_2_O	5.782	4.646	-5.214	0.568	1.759	23.912	0.042	5.214	9.172

### Molecular electrostatic potential (MESP) for glycine/nano metal oxides model molecules

**Figure 3:**
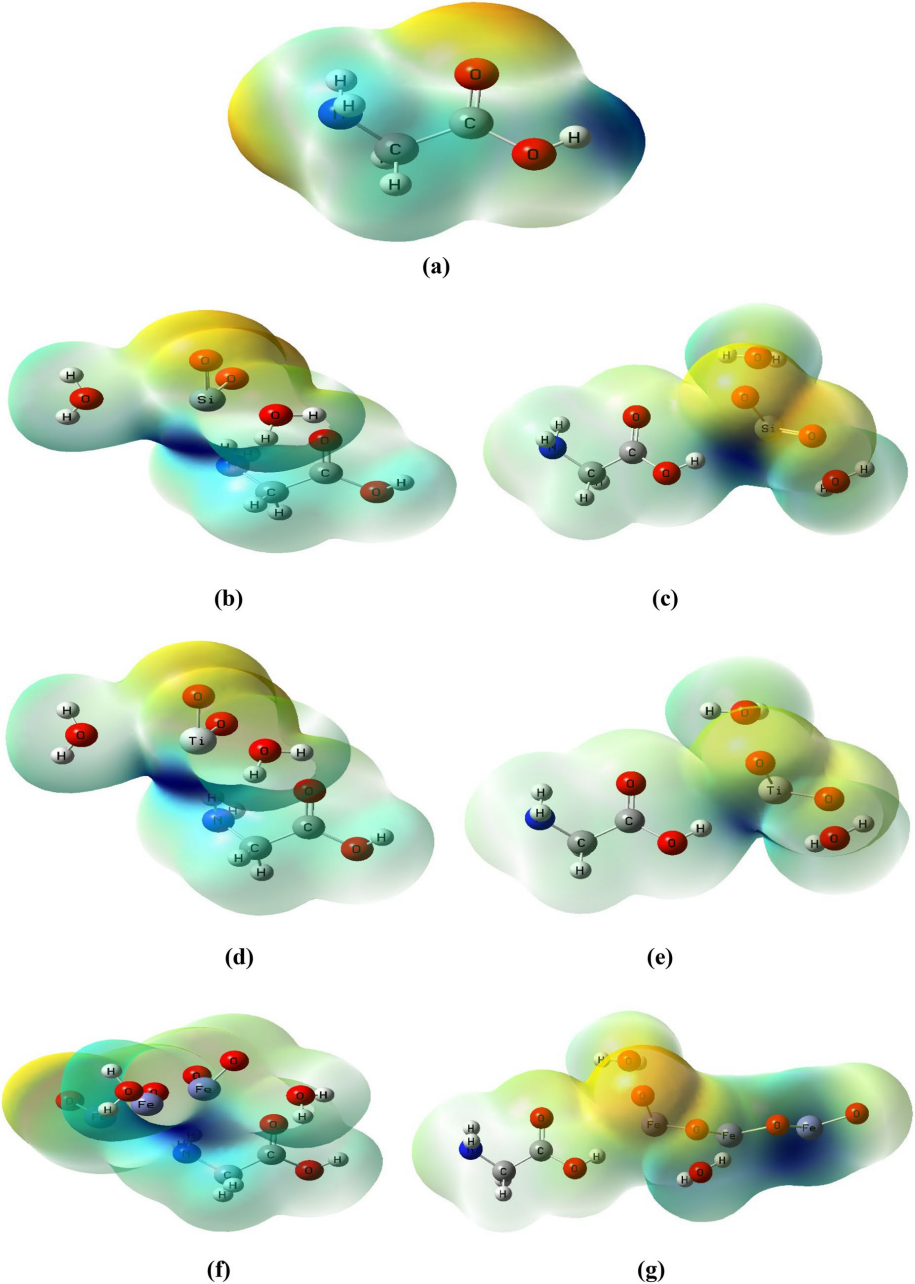
B3LYP/6-31G(d, p) calculated MESP map of glycine two active sides (NH_2_ and OH) interacted with metal oxides including SiO_2_, TiO_2_, and Fe_3_O_4_ in presence of two water molecules.

To gain a better understanding of the electronic density on the molecular surface and to describe the active sites for both electrophilic and nucleophilic reactions as well as hydrogen bonding interactions, 3D plots of the MESP maps of the models under study have been drawn at the water phase (see Fig. 3). The proton is attracted to the concentrated electron density in the molecule (red color) when the electrostatic potential is negative. In areas with low electron density and partial nuclear charge shielding, the positive electrostatic potential (blue color) is associated with the atomic nuclei’s repulsion of the proton. MESP is significant since it indicates molecular size and shape while also charging electrostatic potential regions in terms of color grading. Red < orange < yellow < green < blue is the order in which the potential increases^50–52^. The MESP map shows that, primarily in glycine, the hydrogen atoms of amino and carboxyl groups are located in locations with a positive potential (blue regions). A negative electrostatic potential is present over the carboxyl group’s oxygen atoms and amid group’s nitrogen atoms in the orange-red regions, as presented in Fig. 3-a.

Figure 3-b, c, d, e, f, and g depicted the change in color distribution of glycine after the interaction with SiO_2_, TiO_2_, and Fe_3_O_4_ metal oxides. Low-potential (red to yellow) areas show an abundance of electrons, whereas high-potential (dark blue to light blue) areas show an absence of electrons. As a result, the low-potential (red to yellow) areas indicate high reactivity and the most probable interaction site, susceptible for attack by electrophiles. According to the MESP maps, the color of the surface of the glycine AA changed from light blue to dark blue as a result of metal oxide interaction, which reflects a change in the glycine surface reactivity. Additionally, the figure showed that red- orange regions extended to the metal oxides under study, which confirms the increased reactivity of glycine. The obtained results of MESP are in good agreement with those of ω and N.

### Density of states for (DOS) glycine/nano metal oxides model molecules

**Figure 4:**
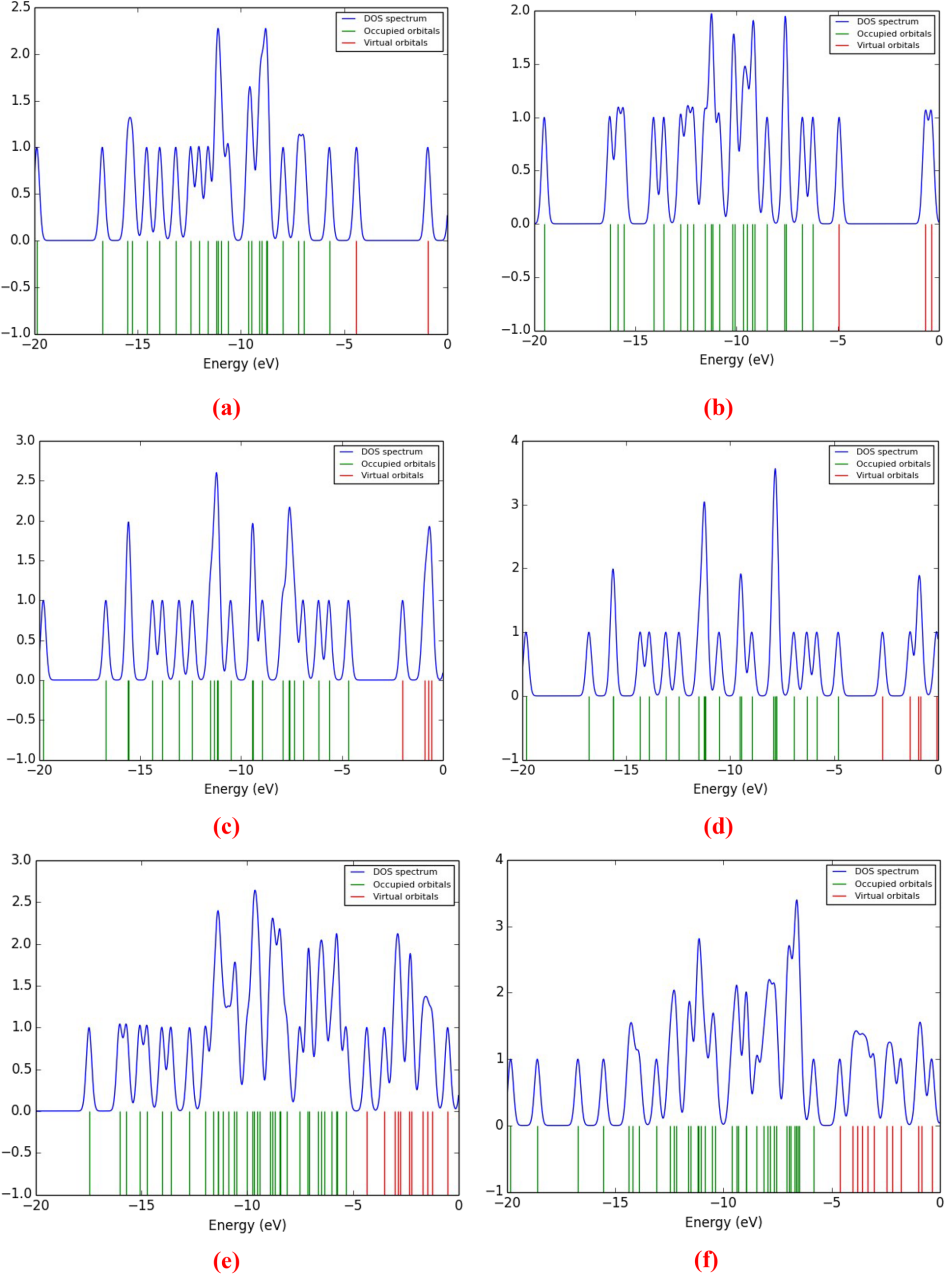
B3LYP/6-31G(d, p) calculated TDOS of glycine interacted with (**a**) and (**b**) SiO_2_, (**c**) and (**d**) TiO_2_, and (**e**) and (**f**) Fe_3_O_4_ in presence of two water molecules through the NH2 and COOH functional groups, respectively .

A DOS analysis is used to examine the electrical characteristics and reactivity of the different structures. We calculated the total DOS (TDOS) and plotted the spectrum between − 20 and 0 eV (Fig. 4). DOS analysis measures the total amount of states per unit energy, with newly generated HOMO states indicating the molecule’s conductivity. The HOMO energy line for glycine appeared at -6.726 eV, while LUMO states appeared at -0.637 eV, as presented in reference^53^. However, the HOMO energy line appeared at -5.742, -6.234, -4.724, -4.764, -5.364, and − 5.825 eV, while LUMO states were near − 4.343, -4.948, -1.998, -2.673, -4.343, and − 4.618 eV for glycine interacted with SiO_2_ (through NH_2_ and COOH groups), TiO_2_, and Fe_3_O_4_, respectively. Higher HOMO energy leads to increased electron transfer to LUMO. This reduces HOMO-LUMO gaps and improves charge transfer speed. Due to the interaction of glycine with metal oxides, the Fermi level rises due to an increase in free carriers near the valence band maximum. Hence, the conduction band minimum shifts towards the Fermi level, narrowing the band gap in the visible electromagnetic spectrum. Accordingly, the studied models show potential for optoelectronic and light-emitting diode (LED) fabrication due to a shift in bandgap caused by metal oxide interaction.

### UV-Vis. analysis for glycine/nano metal oxides model molecules

**Figure 5:**
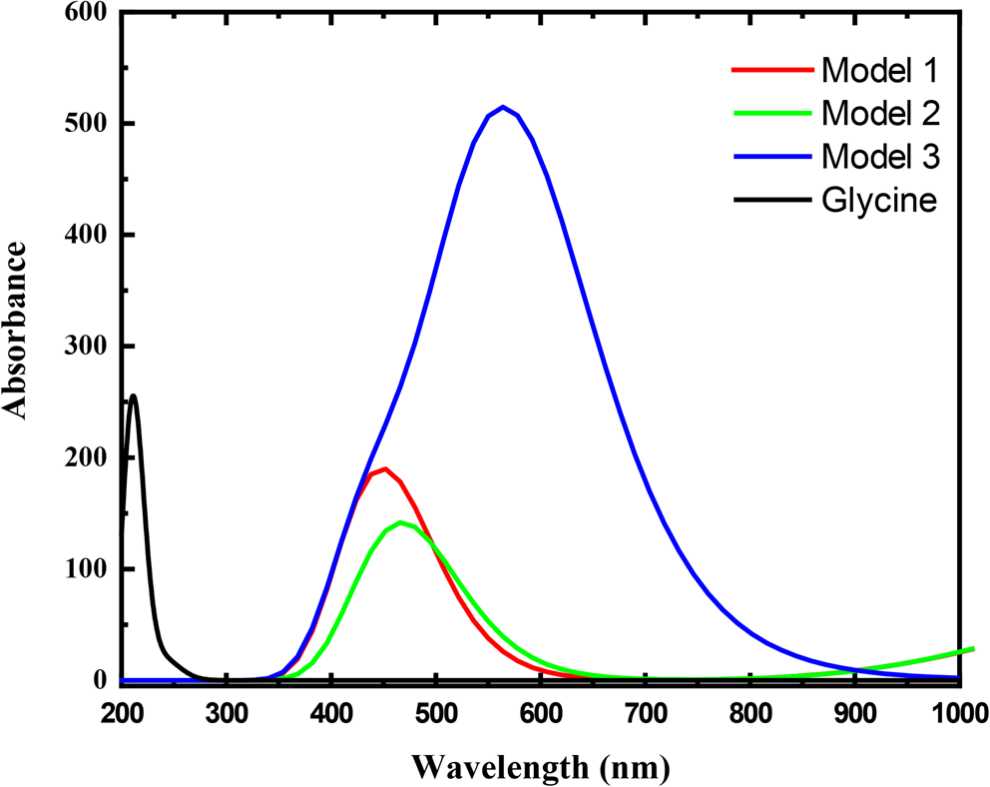
TD-DFT/CAM-B3LYP/6–31G(d, p) calculated UV – vis. absorption spectra of Glycine, Glycine – (COOH) SiO_2_ -H_2_O (Model 1), Glycine (COOH)-TiO_2_-H_2_O (Model 2), Glycine (COOH)-Fe_3_O_4_ -H_2_O (Model 3).

Figure 5 shows the UV-Vis absorption spectra of glycine and glycine interacted with SiO_2_, TiO_2_, and Fe_3_O_4_ calculated at the DFT/CAM-B3LYP/6-31G(d, p) level of theory. Figure 5 shows a red shift in the absorption maxima of glycine due to the interaction with SiO_2_, TiO_2_, and Fe_3_O_4_ through the COOH group associated with an increase in polarity as the absorption intensity increased. As presented in the figure, the characteristic absorption peak of glycine appeared at 212 nm, while that of glycine interacted with SiO_2_, TiO_2_, and Fe_3_O_4_ appeared at 449, 467, and 562 nm, respectively. All bands are associated with the n-π* transitions. This suggests that the excited state becomes more stable as the polarity increases. Additionally, the strong shift in the absorption maxima reflects the strong interaction between glycine and the studied metal oxides and confirms the decrease in the energy gap of glycine. These results are consistent with the FMOs and DOS results.

### TDM and HOMO/LUMO bandgap energy for glycine/SiO2 /heavy metals model molecules

**Figure 6:**
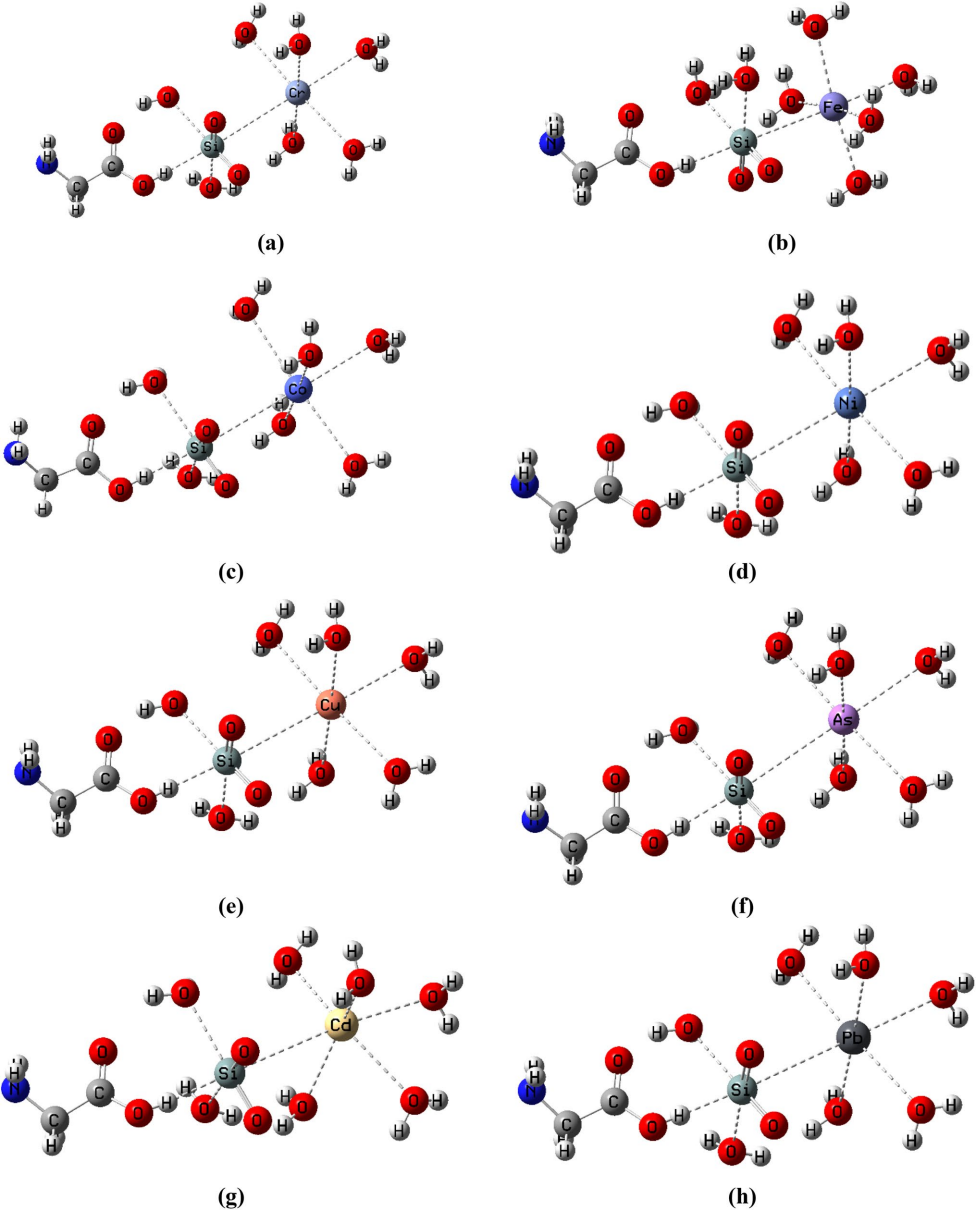
Model structure of glycine/SiO_2_ for hydrated heavy metals removing including (Cr, Fe, Co, Ni, Cu, As, Cd and Pb).

Glycine, a bio-adsorbent with unique hydrogen bonding, has been presented as a metal removal model. Among the studied metal oxides, since the glycine/SiO_2_ model possesses the lowest HOMO/LUMO bandgap energy, SiO_2_ was added to glycine to improve its adsorption affinity. This model involves one glycine monomer and one SiO_2_ monomer interacting with metals like Cr, Fe, Co, Ni, Cu, Zn, As, Cd, and Pb. These heavy metals are surrounded by five water molecules. Glycine/SiO_2_ and the studied heavy metals produce a composite due to hydrogen bonding. Figure 6 depicts the chemical structure of the developed compounds.

Table 4 presents the changes in the values of TDM, HOMO energy, LUMO energy, and HOMO/LUMO bandgap energy of the glycine/SiO_2_ composite due to the adsorption of heavy metals on the surface of the proposed structure of glycine/SiO_2_. The results showed that TDM increased from 7.087 (Debye) to 14.943, 17.426, 11.688, 34.040, 15.780, 10.689, 15.162, and 21.691 Debye due to the adsorption of Cr, Fe, Co, Ni, Cu, As, Cd, and Pb, respectively. Moreover, Table 3 reflects the fluctuations in the HOMO and LUMO energies of glycine/SiO_2_ model molecules due to the adsorption of heavy metals.

The results show that the HOMO and LUMO electron densities of glycine are strongly impacted when it is bound to a heavy metal. As the HOMO energy of the Glycine (COOH)-SiO_2_-H_2_O model molecule changed from − 6.229 eV to -3.684, -3.071, -4.181, -3.747, -3.685, -4.469, -4.283, and − 3.168 eV, while the LUMO changed from − 4.931 eV to -2.790, -2.193, -3.122, -3.499, -2.533, -3.070, -3.176, and − 2.304 eV for Cr, Fe, Co, Ni, Cu, Zn, As, Cd, and Pb, respectively. These fluctuations in both HOMO and LUMO energies reflect that the addition of heavy metals has a more pronounced effect on the HOMO-LUMO gap.

Additionally, the HOMO/LUMO bandgap energy decreased sharply from 1.298 eV to 0.894, 0.878, 0.249, and 0.864 eV due to the adsorption of Cr, Fe, Ni, and Pb, respectively, but it increased to 1.059, 1.152, 1.399, and 1.107 eV due to the adsorption of Co, Cu, As, and Cd, respectively. The changes in the TDM and HOMO/LUMO bandgap energy reflect the high reactivity of the proposed structure of glycine/SiO_2_, highlighting the promising role of glycine/SiO_2_ nanocomposite in wastewater treatment.

**Table 4:** B3LYP/LanL2DZ calculated total dipole moment (TDM) as Debye, HOMO energy, LUMO energy, and HOMO/LUMO bandgap energy ($\:\varDelta\:$E) as eV for glycine/SiO_2_ for removing hydrated heavy metals including (Cr, Fe, Co, Ni, Cu, As, Cd and Pb).

Structure	TDM (Debye)	HOMO (eV)	LUMO (eV)	$\:\varDelta\:$E (eV)
Glycine(COOH)-SiO_2_-H_2_O	7.087	-6.229	-4.931	1.298
Glycine/SiO_2_-Cr	14.943	-3.684	-2.790	0.894
Glycine/SiO_2_-Fe	17.426	-3.071	-2.193	0.878
Glycine/SiO_2_-Co	11.688	-4.181	-3.122	1.059
Glycine/SiO_2_-Ni	34.040	-3.747	-3.499	0.249
Glycine/SiO_2_-Cu	15.780	-3.685	-2.533	1.152
Glycine/SiO_2_-As	10.689	-4.469	-3.070	1.399
Glycine/SiO_2_-Cd	15.162	-4.283	-3.176	1.107
Glycine/SiO_2_-Pb	21.691	-3.168	-2.304	0.864

### Electronic parameters of glycine/SiO2 /heavy metals

Electronic transitions are facilitated by molecules with a soft structure that may readily modify their excited states. The electrical characteristics of the adsorbent are shown in Table 5 after the heavy metals are adsorbed.

Observations showed that after metal adsorption, chemical potential, softness, nucleophilicity index, and electronic charges rose, while ionization energy, electron affinity, chemical hardness, electrophilicity, and electronegativity reduced. The opposite of hardness is softness. It shows the ease with which a system or molecule can adapt its electron density to variations in the electron count. The softness and electrophilicity of nickel and lead are higher than the rest of the studied metals. Additionally, nickel and lead showed a lower chemical hardness than the others. This implies that nickel and lead have a somewhat higher capacity for adsorption with respect to the adsorbent. Furthermore, the stability of the examined complexes is indicated by the negative chemical potential values before and after metal adsorption.

**Table 5:** B3LYP/LanL2DZ calculated: ionization energy IE, Electron affinity (EA), Chemical hardness (η), Chemical potential (µ), Chemical softness (S), Electrophilicity index (ω), nucleophilicity index (N), Electronegativity (χ), and electronic charges (ΔN_max_) for glycine/SiO_2_ for removing hydrated heavy metals including (Cr, Fe, Co, Ni, Cu, As, Cd and Pb).

Structure	IE(eV)	EA(eV)	µ(eV)	η(eV)	S(eV)^−1^	ω(eV)	(*N*)(eV)^−1^	χ(eV)	ΔN_max_
Glycine (COOH)-SiO_2_-H_2_O	6.229	4.931	-5.580	0.649	1.541	23.984	0.042	5.580	8.596
Glycine/SiO_2_-Cr	3.684	2.790	-3.237	0.447	2.237	11.722	0.085	3.237	7.242
Glycine/SiO_2_-Fe	3.071	2.193	-2.632	0.439	2.278	1.520	0.658	2.632	5.996
Glycine/SiO_2_-Co	4.181	3.122	-3.652	0.530	1.888	3.530	0.283	3.651	6.896
Glycine/SiO_2_-Ni	3.747	3.499	-3.623	0.124	8.041	0.816	1.225	3.623	29.133
Glycine/SiO_2_-Cu	3.685	2.533	-3.109	0.576	1.736	2.784	0.359	3.109	5.397
Glycine/SiO_2_-As	4.469	3.070	-3.769	0.699	1.430	4.968	0.20	3.769	5.390
Glycine/SiO_2_-Cd	4.283	3.176	-3.729	0.553	1.807	3.848	0.260	3.729	6.737
Glycine/SiO_2_-Pb	3.168	2.304	-2.736	0.432	2.314	1.618	0.618	2.736	6.332

### DOS of glycine/SiO2 /heavy metals

**Figure 7:**
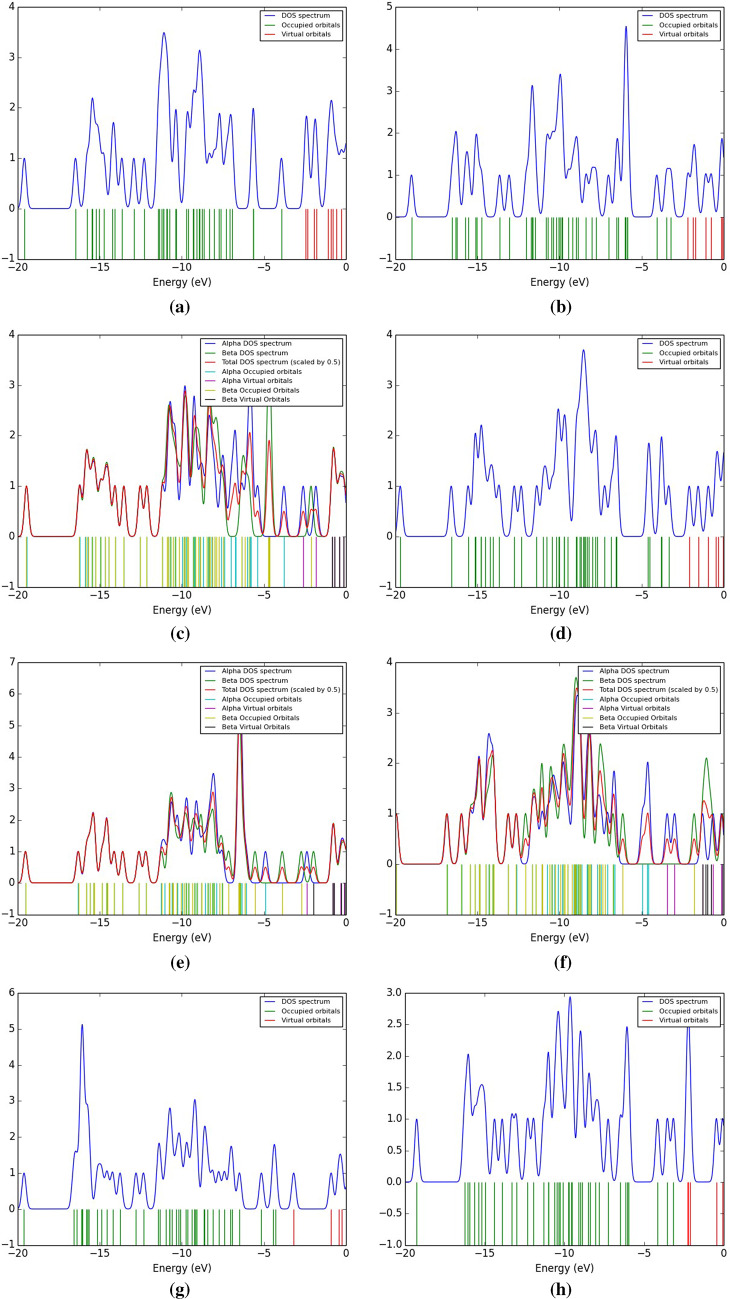
B3LYP/LanL2DZ calculated TDOS of **a**) Cr, **b**) Fe, **c**) Co, **d**) Ni, **e**) Cu, **f**) As, **g**) Cd, and **h**) Pb adsorbed on the glycine - SiO_2_ model molecule.

To understand the adsorption performance of Cr, Fe, Co, Ni, Cu, As, Cd, and Pb on the hydrated Glycine (COOH)-SiO_2_ model molecule’s surface, DOS calculation has been performed in detail. The TDOS of the adsorbed system have been evaluated at the B3LYP/LanL2DZ level, and the calculated results are shown in Fig. 7. The band gap of the hydrated Glycine (COOH)-SiO_2_ model molecule is observed to be different from the Cr, Fe, Co, Ni, Cu, As, Cd, and Pb –adsorbed hydrated Glycine (COOH)-SiO_2_ model molecule surface. As the HOMO energy line appeared at -6.234 eV, while the LUMO line appeared at -4.948 eV for glycine interacted with SiO_2_ through the COOH group. However, the adsorption of Cr, Fe, Ni, and Pb on the surface of the hydrated Glycine (COOH)-SiO_2_ model molecule exhibits a larger variation in the band gap. As the HOMO energy line appeared at -3.884, -3.223, -1.829, -3.297, -2.709, -1.829, -4.287, and − 3.171 eV, while LUMO states were near − 2.453, -2.159, -0.801, -2.086,-1.976, -1.242, -3.186, and − 2.225 eV for the Cr, Fe, Co, Ni, Cu, As, Cd, and Pb -adsorbed hydrated Glycine (COOH)-SiO_2_ model molecule’s surface. It is also noticed from Fig. 7 that the distribution of electrons on the Glycine (COOH)-SiO_2_ model molecule is affected significantly due to the adsorption of Cr, Fe, Ni, and Pb on its surface.

### Combined theoretical and experimental IR spectra

**Figure 8:**
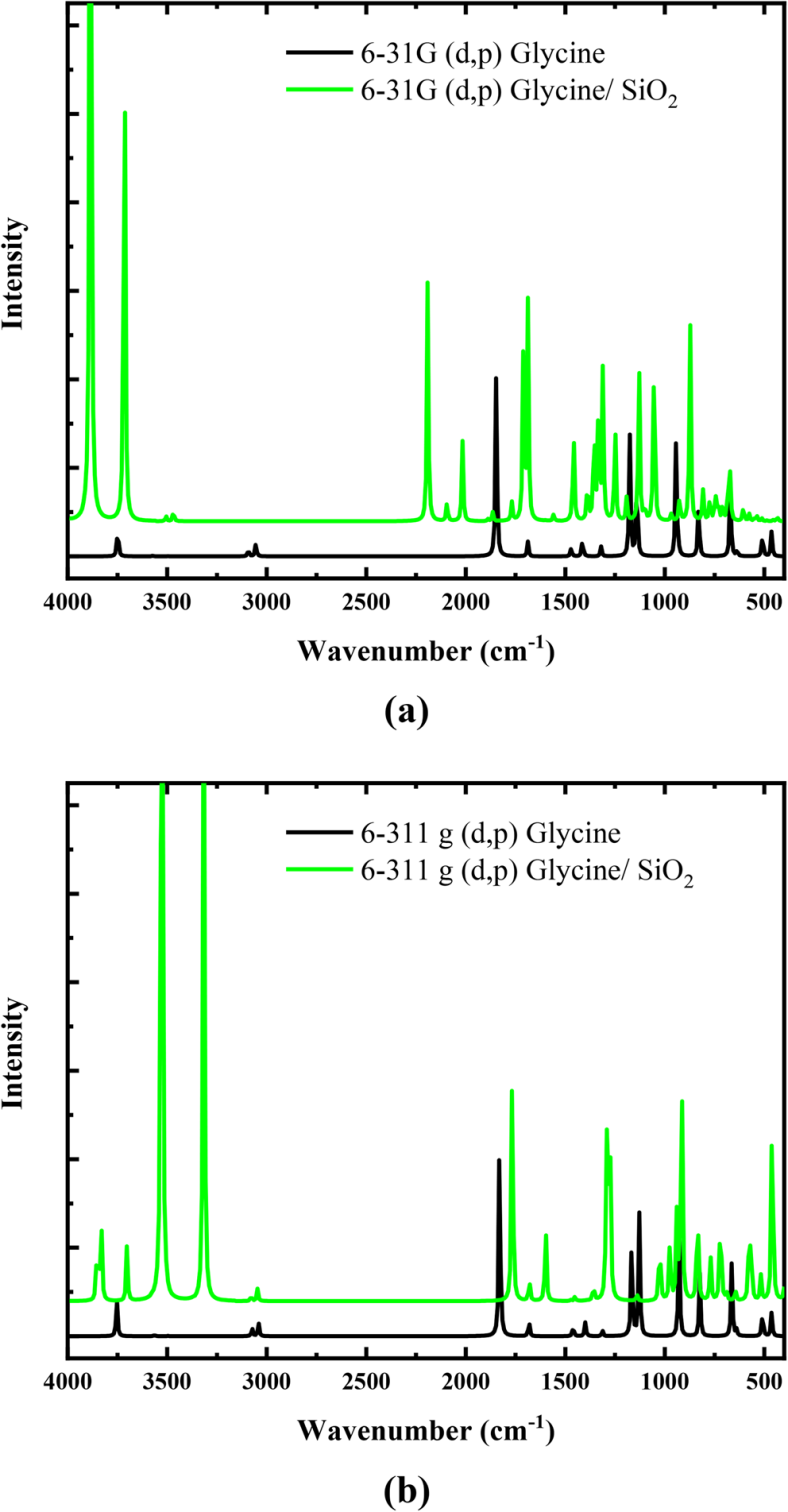
IR spectra of glycine and glycine/SiO_2_ model calculated at **a**) B3LYP/6-31G(d, p) and **b**) B3LYP/6-311G(d, p).

**Figure 9:**
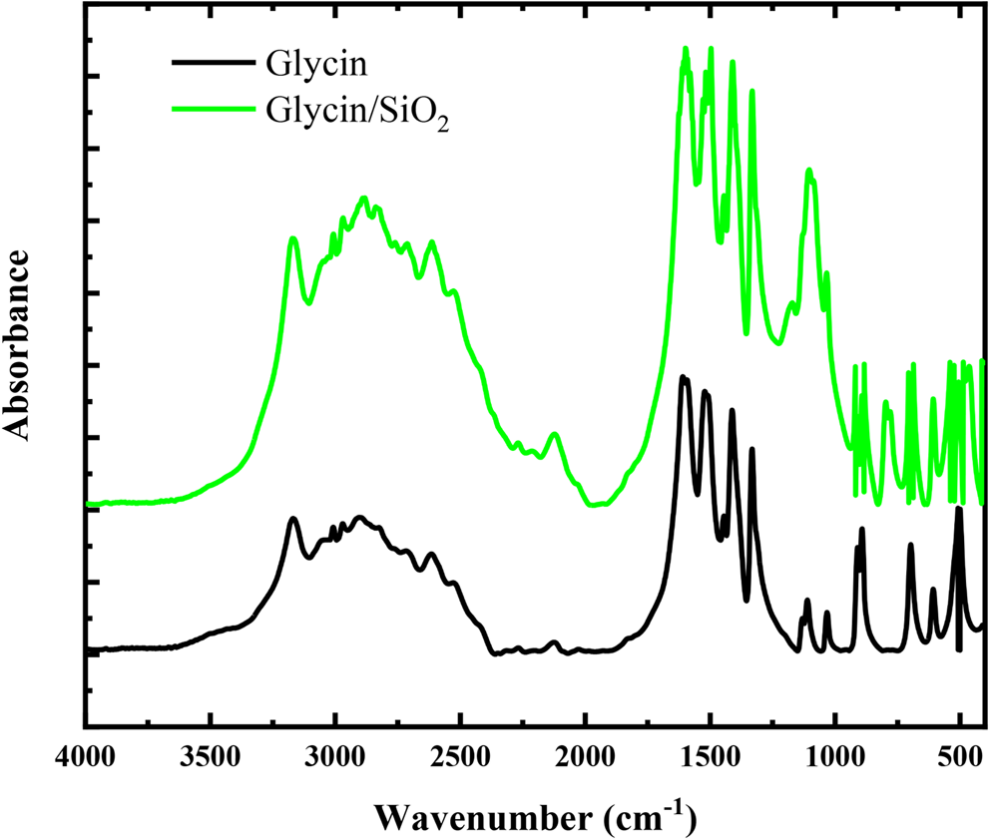
Experimental FTIR spectra of glycine and glycine/SiO_2_ nanocomposite.

In organic chemistry, vibrational spectroscopy is frequently used to study reactions, kinetics, molecular confirmations, and the identification of functional groups in organic materials^54^. The theoretical and experimental FTIR spectra of glycine and glycine/SiO_2_ nanocomposites are presented in Figs. 8 and 9, respectively. Figure 8-a presents the differences in the IR spectra of glycine and glycine/SiO_2_ nanocomposites calculated at the B3LYP/6-31G(d, p) level of theory. Meanwhile, Fig. 8-b presents the differences in the IR spectra of glycine and glycine/SiO_2 _nanocomposites calculated at the B3LYP/6-311G(d, p) level of theory. The B3LYP approach produces geometric parameters that are significantly closer to the experimental data. Thus, we consider both the B3LYP/6-31G(d, p) and B3LYP/6-311G(d, p) levels when discussing glycine vibration modes. The calculated vibrational wavenumbers were uniformly scaled down by a factor of 0.962 for B3LYP/6-31G(d, p) and by a factor of 0.966 for B3LYP/6–311G(d, p) level of theory, which accounts for systematic errors caused by basis set incompleteness, neglecting electron correlation, and anharmonicity of vibrations^55^. Table 6 presents the theoretical (unscaled and scaled) IR band assignment for glycine and glycine/SiO_2_ model molecules in the gas phase.

The unscaled IR spectrum of glycine is carried out at two different basis sets: 6-31G(d, p) and 6-311G(d, p). As tabulated in Table 6, the OH stretching vibration of glycine is observed at 3748 and 3753 cm^−1^ at the basis sets 6-31G(d, p) and 6-311G(d, p), respectively. NH_2_ asymmetrical stretching observed at 3564 and 3572 cm^−1^ for the same sequence. Meanwhile, the CH asymmetrical stretching was observed at 3054 and 3091 cm^−1^ and the CH symmetrical stretching was observed at 3054 and 3041 cm^−1^. The high intensity band observed at 1846 and 1830 cm^−1^, for the same sequency, is assigned to C = O stretching vibration with C-O-H bending and NH_2_ scissoring. NH_2_ bending vibrations observed at 1688 and 1682 cm^−1^. Finally, the observed bands in the region from 942 to 463 cm^−1^ are assigned to NH_2_ + CH_2_ + CO_2 _bending vibrations^55^.

For the Glycine (COOH)-SiO_2_-H_2_O model molecule calculated at the 6-31G(d, p) and 6-311G(d, p), small bands observed at 3840, 3851, 3813, and 3831 cm^−1^ were assigned to the OH stretching vibrations (see Table 7). The intense bands at 3477 and 3306 for the basis set 6-31G(d, p) and 3526 and 3500 cm^−1^ for the basis set 6-311G(d, p) correspond to OH symmetric stretching with little NH_2_ symmetric stretching. Meanwhile, the band observed at 1783 cm^−1^ for the basis set 6-31G(d, p) and 1765 cm^−1^ for the basis set 6-311G(d, p) corresponds to C = O stretching with little C-O-H bending. NH_2_ bending and H-O-H bending with little intensity are observed at 1685 and 1608 cm^−1^ for the basis set 6-31G(d, p) and 1680 and 1599 cm^−1^ for the basis set 6-311G(d, p). Additionally, NH_2_ with CH_2_ bending coupled vibrations with high intensity observed at 1360 and 1358 cm^−1^ for the basis sets 6-31G(d, p) and 6-311G(d, p). C = O stretching with little H-O-H bending observed at 1279 and 1276 cm^−1^ for the same sequence. The bands at 1050 and 1025 cm^−1^ correspond to C-O-H bending, while the bands 947 and 938 correspond to Si = O stretching with Si-O-H bending, but their assignment is not certain. NH_2_ observed again at 926 and 915 cm^−1^ for the same sequence. CO_2_ bending coupled with Si = O stretching vibrations observed with small intensity at 841 and 835 cm^−1^. O = Si = O bending vibration observed at 770 cm^−1^ for the basis set 6-311G(d, p), but it does not appear at the B3LYP/6-31G(d, p) level. Finally, other bands with intermediate intensities were observed at 721, 648, 517, and 464 cm^−1^ for the basis set 6-31G(d, p) and at 720, 575, 517, and 459 cm^−1^ for the basis set 6-311G(d, p). These bands are attributed to the O = Si = O stretching, O = Si = O bending *+* CO_2_ bending, C-C = O bending, and O = Si = O bending + Si-O-H bending. As presented in Table 6, it’s clear that there is a shift between the observed wavenumbers for the glycine and glycine/SiO_2_ models at the two basis sets. Additionally, the results show that the intensity of glycine vibrational bands increased due to the interaction with SiO_2_, which reflects the strong interaction between them.

Moreover, the experimental FTIR absorption spectra of glycine and glycine/SiO_2_ nanocomposite are presented in Fig. 9. The presence of high intensity IR spectrum bands at 3172 and 3008 cm^−1^ in the experimental IR spectrum of glycine is assigned to the formation of OH and NH_2 _stretching vibrations, respectively. It was discovered that these observations matched values found in the literature^55^ and were confirmed by theoretical calculations listed in Tables 6 and 7. The infrared (IR) spectrum is significantly altered by hydrogen bonding interactions, as shown in Figs. 8 and 9, where frequency shifts and increases in IR intensity are associated with the vibrational modes of the functional groups directly involved in the hydrogen-bonded bridges.

CH asymmetrical and symmetrical stretching vibrations were observed at 2970 and 2909 cm^−1^, respectively. Low intensity band assigned to C = O stretching + C-O-H bending + NH_2_ scissoring observed at 1828 cm^−1^. Meanwhile, NH_2_ and CH_2_ bending vibrations were observed at 1609 and 1331 cm^−1^, respectively. Additionally, the C-O-H bending was observed at 1111 cm^−1^. Finally, the bands observed in the region from 910 to 503 cm^−1^ are assigned to NH_2_ + CH_2_ + CO_2_ bending vibrations. Moreover, the functional groups of glycine undergo a shift to the high wavenumber region due to the formation of the glycine/SiO_2_ nanocomposite (see Table 7). As presented in Tables 6 and 7, the theoretically calculated IR spectra of the glycine and glycine/SiO_2_ nanocomposites are in good agreement with the experimental FTIR spectra, which reflects the suitability of the two basis sets for IR frequency calculations and hence validates the proposed structures of the glycine and glycine/metal oxide model molecules.

**Table 6:** The B3LYP/6-31G(d, p) and B3LYP/6-311G(d, p) calculated and experimental IR spectrum of glycine.

Glycine					
Experimental FTIR	Theoretical IR				Assignment
	Unscaled		Scaled		
	6-31g(d, p)	6-311g(d, p)	6-31g(d, p)	6-311g(d, p)	
3172	3748	3753	3605	3625	OH str
3008	3564	3572	3428	3450	NH_2_ a str
2970	3091	3073	2973	2968	CH a str
2909	3054	3041	2937	2937	CH str
1828	1846	1830	1775	1767	C=O str+ C-O-H bending+ NH_2_ scissoring
1609	1688	1682	1623	1624	NH_2_ bending
1331	1413	1460	1359	1410	CH_2_ bending
1132	1185	1186	1139	1145	NH_2_ + CH_2_ wagging
1111	1140	1128, 1165	1096	1089, 1125	C-O-H bending+ NH_2_
910	942	928	906	896	NH_2_ + CH_2_ wagging
889	829	824	797	795	NH_2_ wagging+CO_2_ bending
697	670	670	644	647	NH_2_ + CH_2_ twisting
503	508	508	488	490	NH_2_ + CH_2_ torsion

**Table 7:** The B3LYP/6-31G(d, p) and B3LYP/6-311G(d, p) calculated and experimental IR spectrum of glycine/SiO_2_.

Glycine/SiO_2_ Nanocomposite					
Experimental FTIR	Theoretical IR				Assignment
	Unscaled		Scaled		
	6-31G(d, p)	6-311G(d,p)	6-31G(d, p)	6-311G(d, p)	
3171	3840,3813	3851,3831	3694, 3668	3720, 3700	OH str
3013	3625	3526	3487	3406	OH str+ NH_2_ a str
2977	3477	3500	3344	3381	CH a str
2881	3306	3315	3180	3202	CH str
1723	1783	1765	1715	1704	C=O str + C-O-H bending
1625	1685	1680	1620	1622	NH_2 _bending
1600	1608	1599	1546	1544	H-O-H bending
1331	1360	1358	1308	1311	NH_2_ bending + CH_2_ bending
1171	1279	1276	1230	1232	C=O stretching + H-O-H bending
1033	1050	1025	1010	990	C-O-H bending
922	947	938	911	906	Si=O str+ Si-O-H bending
910	926	915	890	883	NH2 bending
892	841	835	809	806	CO_2_ bending + Si=O stretching
797	-	770	-	743	O=Si=O bending
696	721	720	693	695	O=Si=O stretching
607	648	575	623	555	O=Si=O bending* + *CO_2_ bending
506	517	517	497	499	C-C=O bending
463	464	459	446	443	O=Si=O bending+ Si-O-H bending

### HR-TEM analysis

Recent standards related to the morphology of nanoparticles have been published by the International Organization for Standardization (ISO)’s International Committee on Nanotechnology (TC229). These include ISO 21363:2020, which deals with measuring particle size and shape distributions using a transmission electron microscope, and ISO 19749:2021, which deals with measuring particle size and shape distributions using a scanning electron microscope. The microscope is the only precise tool for measuring particle size since it can directly view and count individual nanoparticles^56^. For each of the three nanomaterial samples, TEM analyses were performed to look into the shape. The TEM images were taken by placing a drop of each solution on a grid made of carbon and copper.

**Figure 10:**
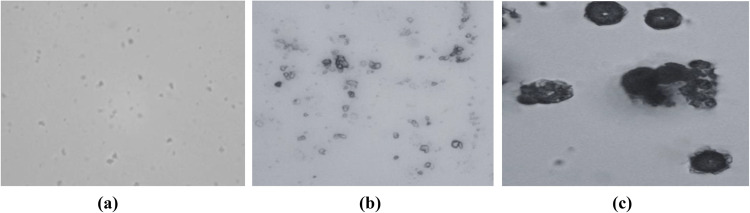
HR-TEM images of **a**) SiO_2_ nanoparticles and **b**) 95%Glycine- 5% SiO_2_NPs, and **c**) 5%Glycine- 95% SiO_2_NPs nanocomposite

TEM images of SiO_2_ nanoparticles indicated that SiO_2_ NPs were spherical without aggregation and well dispersed, as presented in Fig. 10-a. Adsorption of a 5% SiO_2_ nanoparticle on the glycine surface, therefore, showing high dispersion and fewer clusters (Fig. 10-b). Meanwhile, Fig. 10-c represents TEM images of the sample containing 95% glycine and 5% SiO_2_ nanoparticles. The figure confirmed the formation of the nanocomposite with little aggregation of SiO_2_ nanoparticles on the glycine’s surface. Additionally, the results revealed that the addition of glycine amino acids has a substantial effect on the shape of SiO_2_ nanoparticles.

### Antibacterial activity of amino acid-based surfactants

Surfactants that are ideal for industrial development must be (a) multifunctional, (b) low in toxicity, (c) derived from renewable raw materials, (d) biodegradable, and (e) conveniently synthesized. Amino acid-based surfactants comprise long aliphatic chains connected by α-amino, α-COOH, and side chain groups. Surfactants made from amino acids and natural substances have enormous economic and environmental potential since they are environmentally conscious and eco-friendly. Common amino acids utilized for synthesis include glycine, proline, and glutamic acid. These chemicals may be readily transformed into single-chain surfactants by reacting them with hydrophobic chains, including fatty acids, esters, amines, and alcohols^35^.

**Figure 11:**
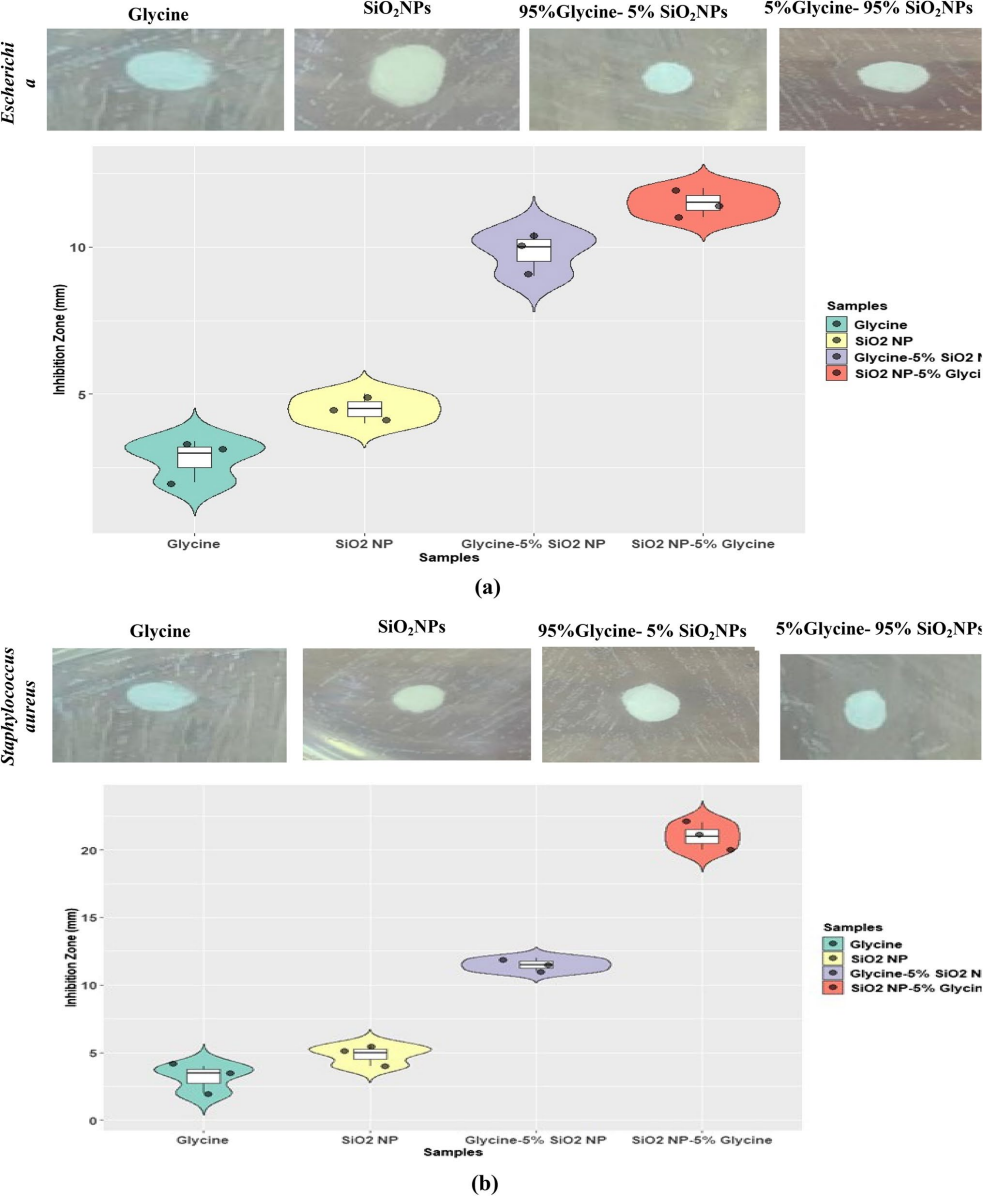
Antibacterial activity of glycine and glycine/SiO_2_ nanocomposites against **a**) *Escherichia coli*and **b**) *Staphylococcus aureus.*

Hydrophobic chains can be incorporated into amino acid components through ester or amide linkages. Amino acids possessing reactive side chains, including glycine, provide new potential for the molecular structure of single-chain surfactants. Single-chain surfactants are advantageous from an economic and environmental standpoint since they may be easily employed as molecules containing only one amino acid within a polar head^35^. As a result, cationic surfactants were made using fatty alcohols or amines to replace eight different kinds of amino acids. Before coating NPs with the produced glycine surfactants, their antibacterial activities were evaluated. The results are summarized in Fig. 11.

We assessed the in vitro antibacterial activity of synthesized glycine against Gram-positive and Gram-negative bacteria that are known to cause human illness. The antibacterial activity was determined by measuring the diameter of the inhibitory zone using the agar diffusion technique. The results of the paper disk assay test revealed that the antibacterial activity was shown in Fig. 11. Glycine, SiO_2_NPs, 95%Glycine- 5% SiO_2_NPs, and 5%Glycine- 95% SiO_2_NPs nanocomposites have antibacterial action against both Gram-positive and Gram-negative bacteria. The sample containing 5%Glycine and 95% SiO_2_ NPs is the more active. 95%Glycine- 5% SiO_2_NPs and 5%Glycine- 95% SiO_2_NPs have more robust antibacterial activity than SiO_2_ NPs alone and glycine alone.

Glycine was enhanced upon being interacted with SiO_2_ NPs. Regarding inhibiting efficiency, apoptotic in *S. aureus* Gram(+ Ve) bacteria was higher than in other *E. coli* Gram(-Ve) bacteria. It is well understood that Gram-negative pathogens are more susceptible to antibacterial drugs than Gram-positive bacteria. This inherent resistance is linked to a lipopolysaccharide outer layer of Gram-negative bacteria, which functions as an effective permeability barrier. However, the bioactivity of nanocomposites can be influenced by a variety of parameters, including chemical structure, morphology, particle size, nanocomposites concentration, and exposure period. As a result, three mechanisms were typically shown to function independently or concurrently: direct attachment to microbe surfaces, metal ion release and penetration into cells, and the generation of reactive oxygen species (ROS). As a result, cell functioning may be disrupted or completely lost, causing cell death.

## Conclusion

Heavy metal ion removal from wastewater is critical for maintaining a clean environment and protecting human health. Several described approaches were used to remove heavy metal ions from various wastewater sources. According to the World Health Organization, we should prioritize environmentally friendly, cost-effective, and sustainable materials and practices. Thus, in this work, DFT calculations were performed at the B3LYP/LanL2DZ level of theory in order to predict the structure and evaluate the glycine/SiO_2_ nanocomposite’s ability to interact with hydrated heavy metals. All proposed molecules’ optimal geometry confirmed the logicality of their structures. The results showed that the TDM increased and HOMO/LUMO bandgap energy decreased due to the interaction of glycine with SiO_2_, TiO_2_, and Fe_3_O_4_. However, the lowest bandgap energy belongs to the glycine/SiO_2_ model when the interaction proceeds through the COOH functional group. The DOS study revealed that more energy levels appeared in the HOMO/LUMO energy of glycine due to the interaction with SiO_2_, TiO_2_, and Fe_3_O_4_ molecules. Moreover, the UV-Vis spectra of glycine and its nanocomposites with SiO_2_, TiO_2_, and Fe_3_O_4_ molecules were studied theoretically using TD-DFT/CAM-B3LYP/LanL2DZ calculations in the gas phase. The UV-Vis absorption spectra of the glycine model molecule red shifted due to interaction with SiO_2_, TiO_2_, and Fe_3_O_4_, resulting in increased absorption intensity. Additionally, band gap energy calculations indicated that Ni, Pb, Fe, and Cr and the glycine/SiO_2_ nanocomposite could interact somewhat more strongly than Co, Cu, As, and Cd. After the complexes interacted with the heavy metals, there was a concurrent increase in softness and decrease in hardness. The softness was reported in the following order: glycine/SiO_2_-As < glycine/SiO_2_ < glycine/SiO_2_-Cu < glycine/SiO_2_-Cd < glycine/SiO_2_-Co < glycine/ SiO_2_-Cr < glycine/SiO_2_-Fe < glycine/SiO_2_-Pb < glycine/SiO_2_-Ni. The results confirmed that the glycine/SiO_2_ nanocomposite could remove all the studied heavy metals; however, nickel had a greater electron affinity for the adsorbent than the others. To summarize, this work provided detailed insights into how glycine and hydrated metals interact. The glycine/SiO_2_ nanocomposite’s efficiency as a bio-adsorbent for removing heavy metals from wastewater was demonstrated. This highlighted the potential of bio-adsorbents for wastewater treatment. Additionally, theoretical and experimental IR spectra confirmed the validity of the structures proposed and the formation of hydrogen bonding between glycine and SiO_2_. HR-TEM images confirmed the distribution of SiO_2_ NP on the glycine surface with little aggregation. Additionally, the results have shown that combining glycine with SiO_2_ NPs can increase the antibacterial activity of glycine. However, more in vivo and clinical studies are needed to show that glycine/SiO_2_ nanocomposites possess antibacterial activities without toxicity.

## Data Availability

The data will be available upon request. Contact Medhat A. Ibrahim, Email: ma.khalek@nrc.sci.eg.
